# Effects of Infantile Hypophosphatasia on Human Dental Tissue

**DOI:** 10.1007/s00223-022-01041-4

**Published:** 2022-11-21

**Authors:** Eva Maria Wölfel, Simon von Kroge, Levi Matthies, Till Koehne, Karin Petz, Thomas Beikler, Carmen Ulrike Schmid-Herrmann, Bärbel Kahl-Nieke, Konstantinos Tsiakas, René Santer, Nicole Maria Muschol, Jochen Herrmann, Björn Busse, Michael Amling, Tim Rolvien, Nico Maximilian Jandl, Florian Barvencik

**Affiliations:** 1 Department of Osteology and Biomechanics, University Medical Center Hamburg-Eppendorf, Lottestr. 59, 22529 Hamburg, Germany; 2 Interdisciplinary Competence Center for Interface Research (ICCIR), Lottestr. 55A, Hamburg, Germany; 3 Department of Oral and Maxillofacial Surgery, University Medical Center Hamburg-Eppendorf, Martinistr. 52, 20246 Hamburg, Germany; 4 Department of Orthodontics, Center for Dental and Oral Medicine, University Medical Center Hamburg-Eppendorf, Martinistr. 52, 20246 Hamburg, Germany; 5 Department of Periodontics, Preventive and Restorative Dentistry, University Medical Center Hamburg-Eppendorf, Martinistr. 52, 20246 Hamburg, Germany; 6 Department of Pediatrics, University Medical Center Hamburg-Eppendorf, Martinistr. 52, 20246 Hamburg, Germany; 7 Department of Diagnostic and Interventional Radiology and Nuclear Medicine, Section of Pediatric Radiology, University Medical Center Hamburg-Eppendorf, Martinistr. 52, 20246 Hamburg, Germany; 8 Division of Orthopaedics, Department of Trauma and Orthopaedic Surgery, University Medical Center Hamburg-Eppendorf, Martinistr. 52, 20246 Hamburg, Germany

**Keywords:** HPP, TNSALP, ALPL, Tooth, Enzyme replacement therapy

## Abstract

Hypophosphatasia (HPP) is an inherited, systemic disorder, caused by loss-of-function variants of the *ALPL* gene encoding the enzyme tissue non-specific alkaline phosphatase (TNSALP). HPP is characterized by low serum TNSALP concentrations associated with defective bone mineralization and increased fracture risk. Dental manifestations have been reported as the exclusive feature (odontohypophosphatasia) and in combination with skeletal complications. Enzyme replacement therapy (asfotase alfa) has been shown to improve respiratory insufficiency and skeletal complications in HPP patients, while its effects on dental status have been understudied to date. In this study, quantitative backscattered electron imaging (qBEI) and histological analysis were performed on teeth from two patients with infantile HPP before and during asfotase alfa treatment and compared to matched healthy control teeth. qBEI and histological methods revealed varying mineralization patterns in cementum and dentin with lower mineralization in HPP. Furthermore, a significantly higher repair cementum thickness was observed in HPP compared to control teeth. Comparison before and during treatment showed minor improvements in mineralization and histological parameters in the patient when normalized to matched control teeth. HPP induces heterogeneous effects on mineralization and morphology of the dental status. Short treatment with asfotase alfa slightly affects mineralization in cementum and dentin. Despite HPP being a rare disease, its mild form occurs at higher prevalence. This study is of high clinical relevance as it expands our knowledge of HPP and dental involvement. Furthermore, it contributes to the understanding of dental tissue treatment, which has hardly been studied so far.

## Background

Hypophosphatasia (HPP, OMIM 146,300, 241,500 and 241,510) is an inherited, systemic, metabolic disorder which manifests clinically in a wide spectrum, including deficient mineralization of bones and teeth resulting in delayed growth, musculoskeletal abnormalities, and poor dental status with premature loss of deciduous and permanent teeth [1, 2]. While the prevalence of severe HPP is 1/300,000, its mild forms have a prevalence of 1/508 [2]. Pathophysiologically, HPP is caused by loss-of-function variants in the *ALPL* gene encoding tissue non-specific alkaline phosphatase (TNSALP) [3], which is a cofactor for numerous enzymatic reactions, dephosphorylates pyridoxal 5′-phosphate (PLP) and hydrolyzes extracellular inorganic pyrophosphate (ePP_i_), a key inhibitor of skeletal and dental mineralization [3–5]. While various forms of HPP may cause hypomineralization of the skeleton, short stature, and waddling gait in infantile forms, osteomalacia, musculoskeletal pain, stress fractures and bone healing disorders occur in adults [6, 7]. Odontohypophosphatasia (OMIM 146,300) is a tooth-specific clinical isotype in which dental tissues are the only affected site with absent skeletal impairments [6–8].

In HPP, next to skeletal and respiratory anomalies, first clinical signs during infancy are deteriorations of the periodontium as well as premature tooth loss without prior root resorption or mineralization defect [9–12]. TNSALP is found in ameloblasts, odontoblasts, cementoblasts and the periodontal ligaments (PDL), suggesting a direct involvement of HPP in hypomineralization, disrupted PDLs, acellular cementum deficiency, and periodontal disease [8, 13–16].

A recent treatment option, primarily for patients with perinatal or infantile HPP, is enzyme replacement therapy (ERT) with asfotase alfa (Strensiq™; Alexion Pharmaceuticals, Inc., USA) [17–20]. Asfotase alfa causes elevated blood activity of TNSALP with the effect of improved respiratory and motor function as well as reduced skeletal symptoms. By this, the osteoid accumulation is decreased, and bone matrix mineralization is improved, reducing fracture risk and improving fracture healing [21, 22]. Treatment efficiency is promising in infants, children as well as adolescents and adults with pediatric onset [23].

Although the beneficial effects of ERT on dental tissues have been described in mouse models of pediatric HPP, reports on humans are scarce and mainly observational [24]. In mice, the microstructure of alveolar bone, acellular cementum and dentin are partially rescued by chimeric and bone targeted alkaline phosphatase, whereas mineralization and thickness of enamel is prevented when injected from the first day following birth [4, 25–27]. Previous case reports of HPP patients have shown, that tooth mobility and premature loss of deciduous teeth can be reduced by treatment with asfotase alfa [28, 29]. However, most studies analyzing asfotase alfa treatment effects lack detailed dental examinations to determine efficacy of treatment on HPP-associated dental-periodontal defects [30]. To evaluate how HPP and treatment with asfotase alfa affects the human dental apparatus, we investigated deciduous teeth of two patients with infantile onset HPP with main emphasis on mineralization pattern and histological changes differentiating between enamel, dentin, and cementum. We hypothesize, that HPP changes dental mineralization in all three dental tissues and postulate that these changes can be improved by ERT.

## Methods

### Sample Collection

To characterize the effect of HPP on dental tissues, eleven exfoliated deciduous teeth (two incisors, three canines, and six molars) were collected from a 24-year-old female patient (patient 1) with a compound-heterozygous mutation in the *ALPL* gene and an infantile onset HPP. Patient 1 presented with muscle weakness, respiratory complications and early loss of deciduous teeth. During adolescence she had suffered from fractures with delayed healing. The detailed patient characterization and description of the musculoskeletal apparatus has been published previously [22]. All teeth exfoliated between the age of 3 and 6 years. For comparison, ten corresponding control deciduous teeth after regular tooth loss (6 to 10 years age) from healthy children of the institute staff were analyzed.

### Case Report of Response to Asfotase Alfa Treatment on Dental Tissue

For investigation of possible effects of asfotase alfa treatment on dental tissues, a 3 years and 2 months old male patient was analyzed (patient 2). He had been diagnosed with HPP because of low serum alkaline phosphatase (ALP) activity with consecutively elevated PLP levels and an increased deoxypyridinoline excretion in urine (Table 1). At the initial presentation he showed a height of 88.5 cm (1st centile), weight of 11.6 kg (3rd centile), and head circumference of 49 cm (8th centile). Further, he presented with an asymmetrical face, muscular hypotonia leading to a waddling gait, delayed motor development, rachitic changes, impaired bone microstructure (Table 1), intermittent abdominal pain, chronic constipation, and early loss of numerous deciduous teeth within the preceding 2 years (nine teeth until the age of 4 years and 8 months). To specify the underlying variant, molecular genetic analysis including gene panel sequencing with reference sequence ENST00000374840 was performed, revealing the missense mutation p.A176T.

**Table 1:** Serum parameters and bone microstructure measured with HR-pQCT of the hypophosphatasia patient (3–4 years) with childhood hypophosphatasia, before and under enzyme replacement therapy (ERT) with asfotase alfa

		Before ERT	Under ERT	Reference
Serum parameters	ALP (U/l)	18 (L)	7692 (H)	159–268
	Bone ALP (µg/l)	5.2 (L)	2439 (H)	68–157
	PLP (µg/l)	697 (H)	31.6 (H)	8.7–27.1
	Calcium (mmol/l)	2.39	2.5	2.14–2.65
	Phosphate (mmol/l)	1.7	1.7	1.22–1.88
	25-OH-D3 (µg/l)	9.2 (L)	31.6	20–79
	PTH (ng/l)	16 (L)	18.9	17–84
	Osteocalcin (µg/l)	–	39.5	8.4–60.7
	DPD/crea (nmol/mmol)	–	23	9.0–25.8

		Before ERT	Under ERT	% of Reference
HR-pQCT	Ct.BMD (mg HA/cm^3^)	644	–	97.3
	Ct. Th (mm)	0.67	–	93.1
	BV/TV (%)	13.7	–	77.0
	Tb.BMD (mg HA/cm^3^)	164.2	–	63.9
	Tb.Th (mm)	0.104	–	155.2
	Tb.N (1/mm)	1.32	–	60.3

Reference values for serum parameters are provided by the local laboratory, reference values for HR-pQCT are provided by Burt et al*.* [31]

At an age of 4 years and 1 month, treatment with asfotase alfa was initiated. Laboratory values were assessed at baseline visit and during asfotase alfa treatment and included ALP, bone ALP, PLP, calcium, phosphate, vitamin D status (25-OH-D3), and parathyroid hormone (PTH). Osteocalcin and desoxypyridinolin (DPD) were assessed during treatment. At baseline high resolution peripheral quantitative computed-tomography (HR-pQCT, XtremeCT I, Scanco Medical, CH) was performed at the right distal tibia in a previously described standardized procedure [31]. By this, cortical bone mineral density (Ct.BMD, mgHA/mm^3^), cortical thickness (Ct.Th, mm), bone volume over tissue volume (BV/TV, %), trabecular thickness (Tb.Th, mm), trabecular number (Tb.N, 1/mm), and trabecular bone mineral density (Tb.BMD, mg HA/mm^3^) were assessed according to previously published guidelines [32].

Shortly prior to initiation of therapy, patient 2 lost one deciduous tooth at the age of 4 years. During asfotase alfa treatment another tooth was lost at the age of 4 years and 8 months. Additionally, two control teeth were collected to match the teeth of patient 2. Clinical data were collected retrospectively, and all procedures performed in this study were approved by the local ethics committee (WF-059/19).

### Sample Preparation

All teeth, *i.e.*, HPP and controls, were halved and dehydrated in an increasing alcohol series. One half was embedded in methyl methacrylate for mineralized tissue assessment using quantitative backscattered electron imaging [33], while the other half was embedded in glycol methacrylate (Technovit 7200, Heraeus Kulzer GmbH, GER) for histological analysis, which were ground to a thickness of 100 µm and subsequently stained with toluidine blue.

### Quantitative Backscattered Electron Imaging (qBEI)

To assess the mineralization degree of enamel, dentin, and cementum of the collected teeth, embedded, co-planar and polished samples were analyzed using a scanning electron microscope (LEO 435 VP, LEO Electron Microscopy Ltd., Cambridge, England) with a backscattered electron detector (BSE Detector Type 202, K.E. Developments Ltd., Cambridge, England) to acquire grey scale images [33]. The following parameters were maintained during the measurement; 680 pA current (confirmed by Faraday cup), 20 mm working distance, 20 keV voltage and ×100 magnification. Grey values were calibrated on aluminum and carbon standard (MAC Consultant Ltd., GB). For each region of interest, enamel, dentin, and cementum, eight images were obtained per region and tooth and evaluated using a custom-made Matlab code (MATLAB R2014a, MathWorks®, USA). Of note, acellular cementum was imaged starting at around 200 µm distal of the enamel-cementum junction to avoid analyzing tissue exposed to deep pockets. To differentiate between cementum and dentin, the regions of interest were selected based on Tomes’ granular layers separating both tissues as well as their different visual appearance. Based on the tissue mineral density distribution five different parameters describing the mineral pattern were determined; (i) the calcium mean representing the average calcium weight percentage (CaMean, wt%), (ii) the calcium peak as the highest calcium weight % observed in the region of interest (CaPeak, wt%), (iii) the standard deviation of the bone mineral density distribution which describes the heterogeneity of the mineralization pattern (CaWidth, wt%), (iv) the percentage of the tooth area with low mineralized tissue regions (CaLow, %) and (v) with high mineralized tissue regions (CaHigh, %). The impact of asfotase alfa on the mineralization degree of cementum and dentin was determined by normalizing the results of patient 2 teeth obtained prior and during treatment to matched control teeth.

### Histology

For the analysis of structural parameters of the dental tissue, histological analysis on toluidine blue stained ground blocks was performed using Osteomeasure (Osteometrix, USA). For the staining, the surface of the tooth was immersed in 10% hydrogen peroxide for 10 min, washed in water and subsequently stained for 30 min using a 1% toluidine blue solution at pH 4.5. The following parameters were obtained; (i) the thickness of the acellular cementum (Ac.cem.Th., µm), (ii) the thickness of the repaired cementum (Repair cem.Th., µm) and (iii) the resorption surface proportional to the measured tooth surface (Resorption S./tooth S., %). To evaluate structural changes of tooth properties under asfotase alfa therapy, the cellular cementum thickness (Cellular Cem. Th., µm), the resorption surface over tooth surface and the dentin thickness (Dentin Th., µm) were determined and normalized to values of control teeth.

### Statistical Evaluation

Data were tested for normality using Kolmogorov–Smirnov test and analyzed using student’s *t* test to compare control teeth with HPP teeth using Graph Pad Prism (Version 9, GraphPad Software, USA). An alpha level below 0.05 was regarded as statistically significant. To determine the influence of asfotase alfa treatment, analysis of tooth extracted during treatment was normalized to values of a matched control tooth. This study confirms to STROBE guidelines (Strengthening the Report of Observational Studies in Epidemiology).

## Results

### Impaired Skeletal and Dental Status of Patients with HPP and Improvement Under Asfotase Alfa

**Fig. 1:**
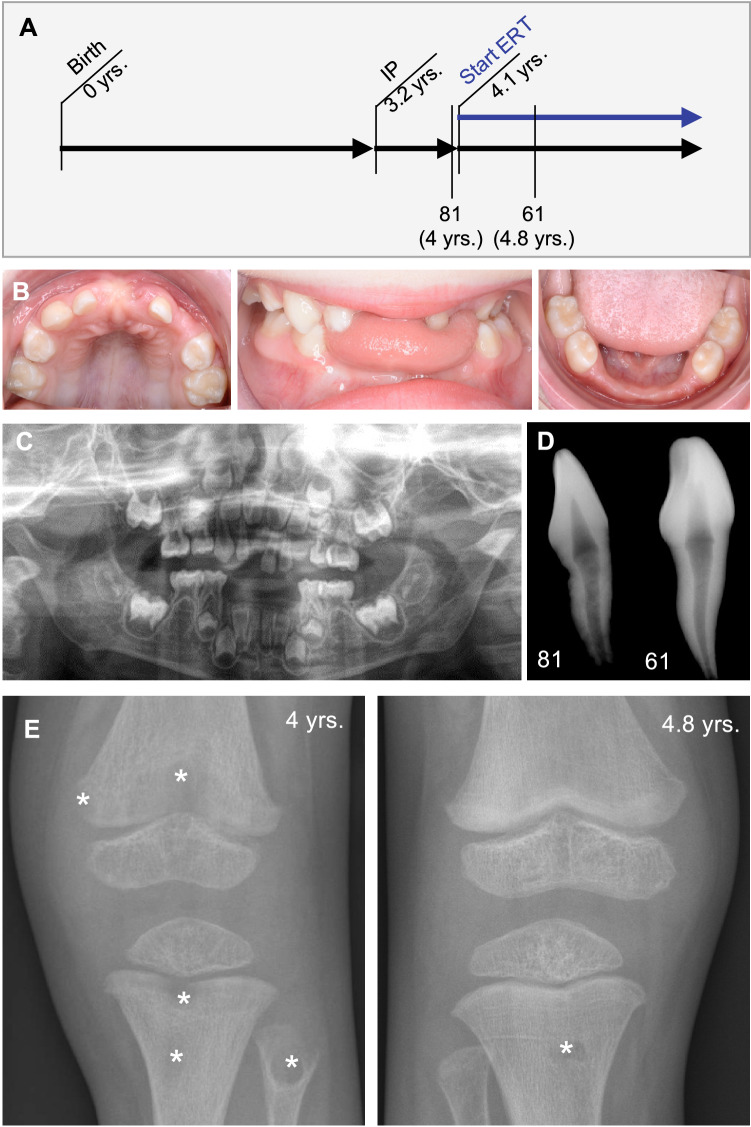
Dental and osteologic status of the male patient 2 before and during ERT with asfotase alfa. **A** Timeline of patient 2 with initial presentation (IP), start of ERT with asfotase alfa (ERT), and exfoliation of both analyzed teeth. **B** Clinical presentation with intraoral photographic images showing normal development of alveolar bone and premature loss of deciduous teeth without signs of periodontal disease or major root resorption at IP. **C** Radiological presentation at IP indicating a rotation and misalignment of the remaining teeth. **D** Radiographic images of lost teeth before (tooth 81) and under (tooth 61) ERT indicating a normal pulp and an intact root with minor signs of resorption only before ERT. **E** On the left panel, radiographic images of left knee before ERT with lesions (*), distended distal metaphases indicating delayed growth. On the right panel, right knee (following 8 months of asfotase alfa treatment) showing an improved bone microstructure. Usage of images were approved by the parents of the patient with written consent

Within this study, teeth from two patients have been analyzed in comparison with matched control teeth. The characterization and skeletal phenotype of patient 1 has already been described in detail including HR-pQCT values [22]. Similar to patient 1, the enzyme replacement therapy with asfotase alfa had a beneficial effect on the skeletal apparatus of patient 2 with infantile onset of HPP. Beginning at age 3, patient 2 suffered from premature loss of his deciduous teeth, while one tooth was also lost during treatment (Fig. 1A). Of note, a right lateral crossbite caused by a narrowed upper jaw was noted, which has potential implications for inappropriate biomechanical stress (Fig. 1B, C). At age 4 years and 8 months (following 1 year of ERT), the exfoliated tooth 61 appeared with less signs of resorption in comparison to the tooth 81, which was lost right before start of the ERT administration. Additionally, the radiographic images indicate a densification and mineralization of trabecular bone near growth plates, *e.g.,* at wrist and knee (Fig. 1E). Serum parameters before and during asfotase alfa treatment confirmed an increase of ALP & bone-specific ALP and a normalization of PTH and PLP, suggesting that mineralization was systemically promoted (Table 1).

### Hypomineralization in Cementum and Dentin of HPP Deciduous Teeth

**Fig. 2:**
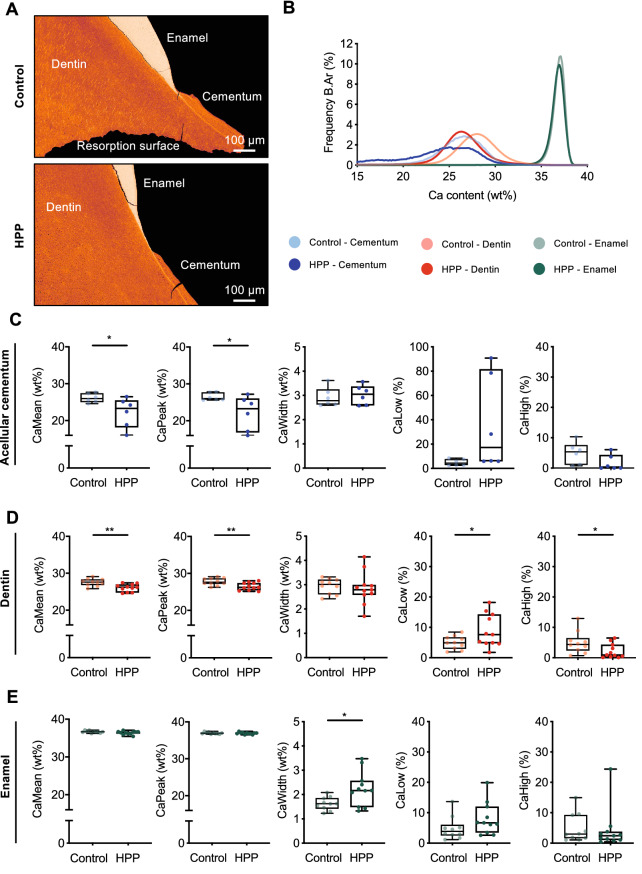
Mineral density distribution in dental tissues of deciduous teeth in a patient with infantile onset hypophosphatasia. **A** Representative color-coded qBEI images of dental tissues acellular cementum, dentin and enamel in control and HPP tooth. High and low mineralized areas are depicted bright and dark red, respectively. **B** Tissue mineral density distribution of dental tissues (acellular cementum—blue, dentin—red, enamel—green) in the HPP patient and control group. **C** Mean (CaMean) and peak (CaPeak) calcium content in cementum of HPP patient 1 was lower compared to control group, whereas the mineralization heterogeneity (CaWidth) as well as the amount of low (CaLow) and high (CaHigh) mineralized areas were similar. **D** In dentin, CaMean and CaPeak of HPP patient 1 was significantly reduced compared to the control group, also a higher CaLow and lower CaHigh was detected. The mineralization heterogeneity was similar between the groups. **E** In enamel, no differences in CaMean, CaPeak, CaLow and CaHigh were detected. The mineralization heterogeneity (CaWidth) was higher in enamel of HPP patient 1 compared to control group

To determine the effect of HPP on the mineralization of cementum, dentin, and enamel, qBEI images were evaluated in teeth from patient 1 and compared to the control cohort (Fig. 2A). The mineral density distributions of the dental tissues indicated differences between both groups in cementum and dentin, whereas enamel of the HPP patient 1 and control group showed a similar distribution (Fig. 2B).

Specifically, the mean (CaMean, 26.1 ± 1.2 wt% *vs.* 21.8 ± 4.3 wt%) and peak (CaPeak, 26.5 ± 1.0 wt% *vs.* 21.6 ± 5.0 wt%) calcium content of the cementum were significantly lower in the HPP patient 1 compared to controls (Fig. 2C), whereas CaWidth (2.9 ± 0.4 wt% *vs.* 3.1 ± 0.4 wt%), CaLow (5.0 ± 2.6% *vs.* 41.9 ± 40.4%), and CaHigh (4.9 ± 3.6% *vs.* 2.1 ± 2.7%) were similar between the two groups.

In dentin, a comparable reduced mineralization was observed with a significantly lower CaMean (27.5 ± 1.0 wt% *vs.* 25.9 ± 1.0 wt%) and CaPeak (27.8 ± 1.0 wt% *vs.* 26.4 ± 1.0 wt%), while CaLow (5.0 ± 2.2% *vs.* 9.3 ± 5.5%) and CaHigh (5.0 ± 3.6% *vs.* 2.0 ± 2.4%) showed a higher amount of hypomineralized regions with concurrent smaller amount of hypermineralized regions. The mineralization heterogeneity (CaWidth, 2.9 ± 0.3 wt% *vs.* 2.9 ± 0.7 wt%) was similar in HPP patient 1 and control group.

In contrast to cementum and dentin, in enamel the CaMean (36.7 ± 0.3 wt% *vs.* 36.3 ± 0.4 wt%) and CaPeak (36.9 ± 0.3 wt% *vs.* 36.8 ± 0.2 wt%) were similar between groups, while CaWidth (1.6 ± 0.3 wt% *vs.* 2.2 ± 0.7 wt%) was significantly higher in the HPP group. CaLow (4.9 ± 3.8% *vs.* 8.5 ± 5.3%) and CaHigh (4.9 ± 4.7% *vs.* 2.3 ± 2.6%) of enamel were similar in both groups.

### Higher Repair Cementum Thickness in HPP Deciduous Teeth

**Fig. 3:**
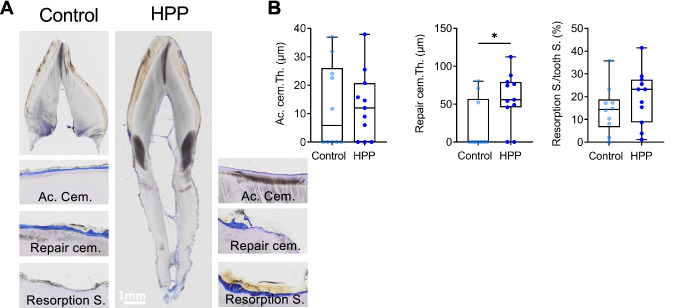
Histologic effects of infantile onset hypophosphatasia on dental tissues of deciduous teeth. **A** Representative images of toluidine blue stained ground tooth samples for Control and HPP showing acellular cementum, repair cementum and resorption surface. **B** Acellular cementum thickness was similar in HPP patient 1 and control, whereas the repair cementum thickness was significantly higher in HPP patient 1 compared to control. The resorption surface over tooth surface presented with similar values for both groups

To analyze the differences in tooth structures, histological evaluation in ground sections was performed. The entire teeth for control group and HPP patient 1 (Fig. 3A) were analyzed by evaluating unique structures of the cementum. Acellular cementum thickness was determined and showed similar values for HPP and control group (17.68 ± 10.26 µm *vs.* 21.20 ± 13.49 µm, respectively). Of note, four out of ten control teeth and two out of eleven HPP teeth showed absent acellular cementum. However, the absence of acellular cementum in control teeth was due to regular complete resorption of the tooth root, whereas in HPP teeth acellular cementum was absent in the above-mentioned cases despite existing tooth roots. A significantly higher repair cementum thickness was observed in HPP teeth compared to control teeth (38.61 ± 5.47 µm *vs.* 3.92 ± 8.66 µm, respectively), which was observed in three out of ten control teeth and in nine out of eleven HPP teeth. The resorption surface over tooth surface in HPP teeth was similar in HPP with 19.69 ± 11.91% compared to control teeth with 14.30 ± 10.36% (Fig. 3B).

### Enzyme Replacement Therapy on Dental Tissue Mineralization and Structure

**Fig. 4:**
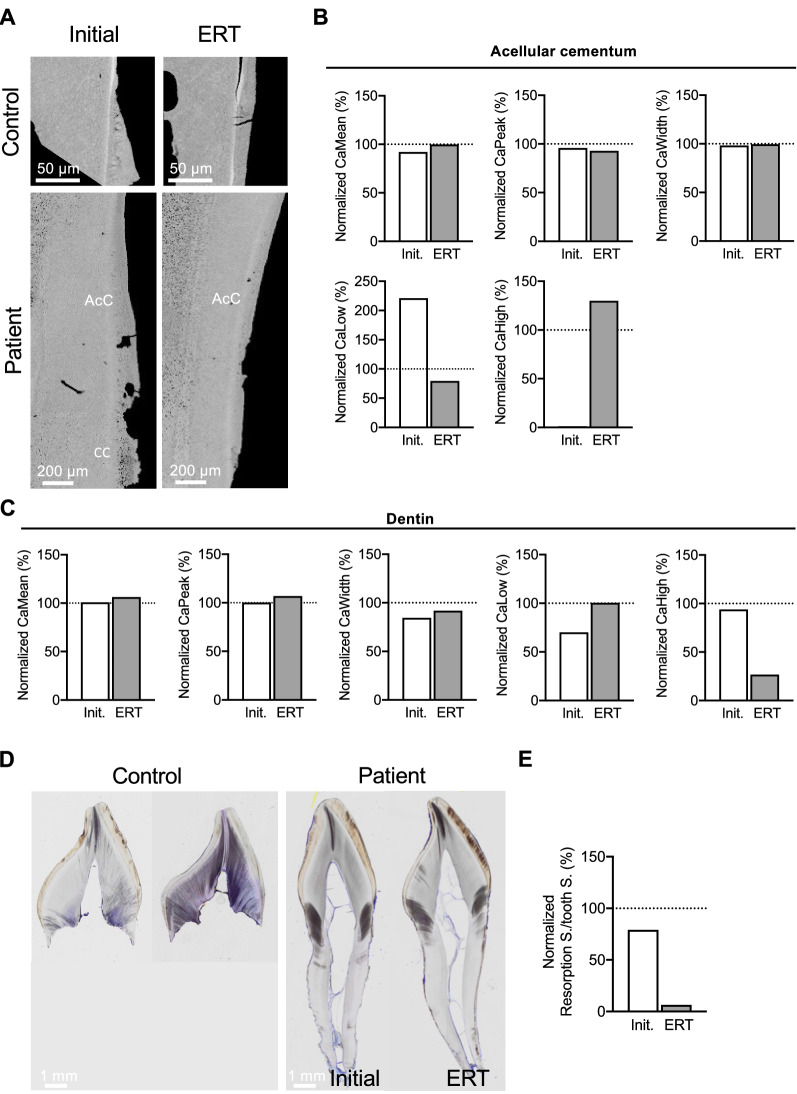
Effects on dental tissues of patient 2 before and during ERT with asfotase alfa in comparison to control teeth. All data from patient 2 were normalized to a control tooth with same age and position. **A** QBEI images of control and patient 2 teeth before and during ERT showing cellular cementum only in the patient before ERT, while simultaneously resorption is present. *AcC* acellular cementum, *CC* cellular cementum. **B** The cementum was hypomineralized before treatment, which adapted to control level with treatment. CaWidth was in range of the control (100%). **C** CaMean and CaPeak of dentin were on control level before treatment and increased during ERT while the mineralization heterogeneity adapted, including increase of CaLow to 100% and a decrease of CaHigh to 27% of the control. **D** Histological changes of dental tissues in patient 2 before and during ERT with asfotase alfa in comparison to control teeth. Overview images of toluidine blue stained teeth (incisors) from patient 2 and matched control teeth before and during asfotase alfa treatment. **E** Normalized resorption surface over tooth surface was reduced in the patient from 79 to 6%

QBEI measurements before and during treatment with asfotase alfa were used to analyze its effect on the three dental tissues, *i.e.,* cementum, dentin, and enamel, in deciduous teeth (Fig. 4A–C). In the tooth of patient 2, cementum mineralization slightly adapted to control level by a reduction of CaLow (221% to 79% normalized to the control) and elevation of CaHigh (1% to 130% normalized to the control). The dentin mean and peak mineralization of patient 2 slightly changed from control level to 106% and 107%. Simultaneously, hypomineralized areas became more and hypermineralized areas less frequent. Of note, changes in mineralization pattern of enamel were not observed.

Regarding histological analysis (Fig. 4D), the resorption surface per tooth surface of patient 2 was reduced during treatment from 79 to 6% normalized to control, while the normalized dentin thickness showed slightly higher values during asfotase alfa treatment in patient 2 (89% and 92%; before and during treatment, respectively; data not shown) (Fig. 4E). Cellular cement thickness was not detectable in patient 2 and matched control tooth.

## Discussion

HPP is a genetic disease that leads to hypomineralization in mineralized tissues due to the lack of cleavage of phosphate from pyrophosphate. Apart from supportive and symptomatic treatment, enzyme replacement therapy with asfotase alfa is the proposed therapy with reported positive effects on the skeleton [19, 34]. Dental hard tissues are mineralized to distinct degrees. Herein, enamel consists of 93–98% inorganic apatite crystals, whereas dentin has an inorganic component of approximately 70%. Root cementum has the lowest mineral content of the dental hard tissues of approximately 65% [35]. The effects of HPP and asfotase alfa treatment on the dental apparatus are still poorly defined. Here, we examined enamel, dentin, and cementum of an infantile onset HPP patient (patient 1) in comparison to age-matched corresponding teeth of healthy controls at the histological and mineralization level and analyzed the effects of asfotase alfa treatment in a child (patient 2).

Our study confirms, that lost teeth in children with HPP generally exhibit substantial remaining root structure compared to healthy control teeth with physiologically resorbed roots [16]. Disorientation of periodontal ligaments and absence of acellular cementum are known detrimental implications on the periodontal apparatus, which facilitate premature loss of teeth [15]. In addition, we were able to show that the mineralization of acellular cementum is disturbed in HPP, indicating an impaired function of cementoblasts, which form and mineralize cementum, causing a weakened attachment site for periodontal ligament at the tooth. Furthermore, we found hypomineralization in dentin of HPP deciduous teeth which correlates with analyses in vitro and in mouse models indicating that odontoblasts can be inhibited in an environment with low ALP activity [27, 36]. Previous studies of exfoliated teeth reported various findings including hypomineralization of dentin, only in mantle dentin or even normal mineralization in HPP patients [11, 16, 37, 38]. In accordance with two studies described effects of infantile HPP, as presented here, while the other two represent findings of childhood and odontohypophosphatasia. This recapitulates the wide range of effects in different forms of HPP showing that mineralization of various dental tissues may be affected. Of note, differing degrees of mineralization may also be reported due to the assessment using distinct modalities varying in dimension (2D *vs* 3D), device dependent resolution, and reported mineralization (calcium weight percentage *vs.* hydroxyapatite).

The acellular cement thickness presented with overall higher values than reported previously, where 2–7 µm thickness in 3 out of 28 HPP teeth were observed [11]. In our collective, up to 37 µm acellular cement thickness in 8 out of 11 HPP teeth was observed. We only found cellular cementum in one HPP tooth and two control teeth, however, others observed cellular cementum in 7 out of 22 HPP teeth [11]. Of note, two of the reported HPP patients had odontohypophosphatasia, suggesting a distinct effect. Interestingly, Kramer et al*.* described acellular cementum in all control teeth and in two HPP canines, while in four incisors, one canine, and one molar no acellular cementum was apparent [38]. Here, we only found acellular cementum in two control incisors and in six HPP teeth, two canines, two incisors and two molars. The absent cementum in control teeth can be explained by the absence of root structure due to the origin of physiological deciduous tooth loss, while HPP teeth were lost with remaining root structure. Absence of acellular cementum is suggested to result in loss of periodontal attachment to the tooth root surface leading to premature exfoliation. As previously described by Kramer et al., no differences of acellular cementum thickness were observed in this study indicating a different mechanism, *e.g.*, low mineralization or ruptured PDL, causing an early exfoliation [38]. We found a significantly higher repair cement thickness in HPP teeth from patient 1 compared to matched control teeth, which is most likely based on the absent root structure in control teeth where resorption repair would be expected.

The 3-year-old male patient 2 presented with premature deciduous tooth loss, low ALP serum levels and impaired bone mineralization allowing for clear diagnosis of HPP. The observed missense variant p.A176T had been reported previously and is of maternal origin [39]. The clinical symptoms and the fact that his parents both presented with reduced ALP plasma activity despite the fact that p.A176T is described as a mild allele with absence of a dominant negative effect [40], suggests that patient 2 carries a second allele which could not be determined yet. This is supported by the observation that a similar phenotype was observed in a compound-heterozygous individual with prenatal manifestations of HPP and his brother who was first evaluated at 19 months of age [41].

Although treatment with asfotase alfa appeared to have beneficial effects on tooth mineralization and phenotypical appearance, the improvement was less pronounced than in the skeleton. As dental tissues lack remodeling and present with a limited formation of acellular cementum and dentin [42], this less pronounced effect is not surprising. Patient 2 lost teeth prematurely, assuming that the connection between acellular cementum and alveolar bone was not sufficiently promoted. Although positive effects of ERT on dental status have been reported in different case reports with significant improvement of teeth mobility [28], loss of already mobile teeth at the start of ERT could not be prevented in one case of odontohypophosphatasia [29]. ERT in *Alpl*^*−/−*^ mouse models before tooth eruption corrected mineralization of bone and teeth as well as mineralized acellular cementum [4, 43]. During childhood administration, the latter may help to prevent accelerated deciduous tooth loss. However, as the administration period of asfotase alfa in patient 2 was relatively short in relation to the mouse studies, longer treatment periods and an earlier initiation will have to be studied to support this assumption. Nevertheless, patient 2 exhibited slightly higher dentin thickness normalized to control teeth during treatment. Dentin has been shown to exhibit improved mineralization during early ERT administration in *Alpl*^*−/−*^ mice [4], which we also found in a slight overall adaptation of acellular cementum and dentin mineralization to control levels in patient 2.

Despite interesting findings, we acknowledge limitations of this report. The main limiting factor is the small sample size and inclusion of various teeth, such as incisor, canine, and molar teeth, from one individual with infantile HPP onset. However, this study also provides the opportunity of quantitatively analyzing the influence of HPP and asfotase alfa treatment on human dental tissues giving a deeper insight into the systemic effects of both. Furthermore, selection of matching control teeth for the patients was difficult due to the pathophysiological nature of HPP premature tooth loss with remaining root structure. Therefore, HPP samples have an intact root structure while control teeth have resorbed roots.

## Conclusion

In conclusion, we here demonstrated that the dental manifestations of infantile HPP are heterogenous. We showed that HPP was associated with detrimental effects on the mineralization and morphology of cementum and dentin while enamel was only slightly affected. Although treatment with asfotase alfa improved skeletal mineralization, beneficial effects on the periodontium are minor. To specify, these results show first data on effects of HPP and of short time treatment with asfotase alfa on dental tissues which are of high clinical relevance to enhance our knowledge on HPP and its treatment on dental tissue. However, future studies are needed to include different forms of HPP, focus on the connection of cementum and alveolar bone and investigate the development of permanent teeth which have at least partially developed during treatment with asfotase alfa.

## Data Availability

All data needed to evaluate the conclusion in the paper are presented in the paper. The datasets generated and/or analyzed during the current study are not publicly available but are available from the corresponding author upon reasonable request.

## Abbreviations

ALP: Alkaline phosphatase
BV/TV: Bone volume over tissue volume
CaHigh: Calcium high
CaLow: Calcium low
CaMean: Calcium mean
CaPeak: Calcium peak
Cellular Cem. Th.: Cellular cementum thickness
Ct.BMD: Cortical bone mineral density
C.Th.: Cortical thickness
Dentin Th.: Dentin thickness
DPD: Desoxypyridinolin
ePP_i_: Extracellular inorganic pyrophosphate
ERT: Enzyme replacement therapy
HPP: Hypophosphatasia
HR-pQCT: High resolution peripheral quantitative computed-tomography
25-OH-D3: Vitamin D status
PDL: Periodontal ligaments
PLP: Pyridoxal 5′-phosphate
PTH: Parathyroid hormone
qBEI: Quantitative backscattered electron imaging
Repair cem.Th.: Thickness of the repaired cementum
Resorption S./tooth S.: Resorption surface proportional to the measured tooth surface
Tb.BMD: Trabecular bone mineral density
Tb.N: Trabecular number
Tb.Th: Trabecular thickness
TNSALP: Tissue non-specific alkaline phosphatase
